# PDGFRβ in Stem Cell Microenvironments: Cross‐Tissue Evidence and Hypotheses for the Limbal Stem Cell Niche

**DOI:** 10.1155/sci/1536168

**Published:** 2026-09-22

**Authors:** Shuying Liao, Guigang Li

**Affiliations:** ^1^ Department of Ophthalmology, Tongji Hospital Affiliated to Tongji Medical College, Huazhong University of Science and Technology, Wuhan 430000, Hubei Province, China, hust.edu.cn; ^2^ Hubei Key Laboratory of Otorhinolaryngologic and Ophthalmic Diseases, Tongji Hospital Affiliated to Tongji Medical College, Huazhong University of Science and Technology, Wuhan 430000, Hubei Province, China, hust.edu.cn

**Keywords:** extracellular matrix remodeling, limbal stem cell niche, PDGFRβ, pericytes, stem cell niche

## Abstract

Stem cells reside within specialized microenvironments, termed niches, composed of vascular networks, stromal cells, extracellular matrix (ECM), immune components, and neural elements, which collectively regulate quiescence, self‐renewal, and lineage commitment of stem cells. Platelet‐derived growth factor receptor‐β (PDGFRβ), a class III receptor tyrosine kinase (RTK) predominantly expressed in perivascular and mesenchymal stromal cells, has been associated with vascular–stromal organization across multiple stem cell systems. Rather than acting mainly as a direct determinant of stemness‐associated transcriptional programs, PDGFRβ may influence stem cell behavior indirectly by modulating vascular stability, matrix remodeling, metabolic gradients, and biomechanical signaling. Evidence from hematopoietic stem cells (HSCs), mesenchymal stem cells (MSCs), neural stem cells (NSCs), tumor stem cells (TSCs), renal glomerular cells, and limbal stem cells (LSCs) indicates that PDGFRβ^+^ stromal or perivascular cells contribute to niche architecture and function in a context‐dependent manner. Through interactions with platelet‐derived growth factor‐B (PDGF‐B), vascular endothelial growth factor (VEGF), hypoxia‐related signaling, inflammatory mediators, and integrin‐dependent mechanotransduction, PDGFRβ is linked to angiogenesis, stromal cell activation, ECM remodeling, and niche biomechanics. Under physiological conditions, such regulation may support tissue maintenance, repair, and regeneration, whereas persistent or dysregulated activation may contribute to fibrosis, vascular dysfunction, tumor progression, and disruption of stem cell equilibrium. This review summarizes the current understanding of PDGFRβ as a vascular–stromal component associated with stem cell niche regulation, with emphasis on cross‐tissue mechanisms and context‐dependent differences. Particular attention is given to the LSC niche, where PDGFRβ^+^ limbal niche cells (LNCs) are discussed in relation to the Palisades of Vogt, limbal vessels, basement membrane/ECM organization, and epithelial progenitor maintenance. We distinguish established evidence for PDGFRβ expression and LNC‐associated stromal identity from proposed mechanisms involving vascular–stromal support, ECM remodeling, biomechanical regulation, and LSC repair. Because receptor‐specific functional evidence for PDGFRβ in limbal microenvironmental cells remains limited, the limbal component of this review is presented as a hypothesis‐generating framework rather than as established evidence that PDGFRβ functionally regulates the limbal niche. Collectively, available evidence supports PDGFRβ as a candidate link between vascular–stromal organization and stem cell functional states, while underscoring the need for direct PDGFRβ perturbation studies in LNCs to define its functional relevance in limbal niche stability and corneal epithelial regeneration. This framework may guide future studies of PDGFRβ‐associated stromal regulation in corneal epithelial regeneration and LSC niche dysfunction.

## 1. Review Methodology and Evidence Appraisal

**Figure 1:**
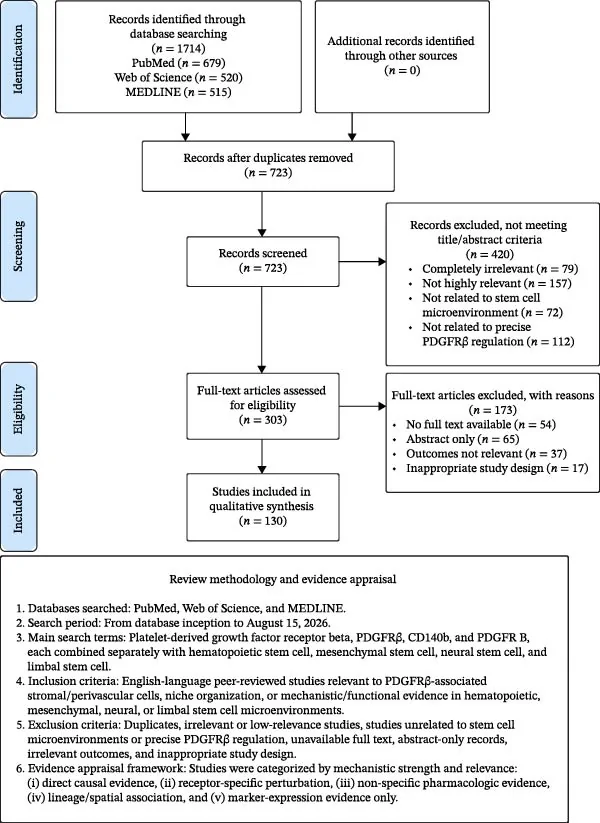
Literature selection and evidence appraisal workflow. A total of 1714 records were identified from PubMed, Web of Science, and MEDLINE. After duplicate removal, screening, and full‐text eligibility assessment, 130 studies were included in the qualitative synthesis and categorized according to mechanistic relevance to PDGFRβ‐associated stem cell microenvironments.

To address the broad and heterogeneous nature of the literature included in this review, we performed a structured literature search and evidence appraisal (Figure 1). PubMed, Web of Science, and MEDLINE were searched from database inception to August 15, 2026. The main search terms included “platelet‐derived growth factor receptor beta,” “PDGFRβ,” “CD140b,” and “PDGFR B,” combined with “hematopoietic stem cell,” “mesenchymal stem cell,” “neural stem cell,” and “limbal stem cell.” English‐language, peer‐reviewed studies were considered eligible if they provided evidence related to platelet‐derived growth factor receptor‐β (PDGFRβ)‐associated stromal or perivascular cells, niche organization, or mechanistic and functional regulation in stem cell microenvironments. Studies were excluded if they were duplicates, irrelevant or only marginally relevant, unrelated to stem cell microenvironments or PDGFRβ regulation, unavailable as full text, abstract‐only publications, or had inappropriate study designs.

Because the available evidence differed substantially across tissues, studies were further appraised according to their mechanistic strength and relevance. Evidence was categorized as direct causal evidence, receptor‐specific perturbation evidence, nonspecific pharmacological evidence, lineage or spatial association, or marker‐expression evidence only. This framework was used to distinguish experimentally supported mechanisms from correlative observations and to guide the interpretation of PDGFRβ function across hematopoietic, mesenchymal, neural, and limbal stem cell (LSC) niches.

## 2. Introduction

PDGFRβ is a class III receptor tyrosine kinase (RTK) that has been implicated in tissue development, vascular maturation, and stromal organization [1]. Since its identification by Yarden and colleagues in 1986, PDGFRβ has been recognized as a receptor for platelet‐derived growth factor‐B (PDGF‐B) and platelet‐derived growth factor‐D (PDGF‐D), which activate the receptor through ligand‐induced dimerization and tyrosine phosphorylation [2–4]. Although PDGFRβ can activate canonical signaling pathways, including phosphoinositide 3‐kinase (PI3K)–Akt, Ras–MAPK, Src‐family kinase, and STAT‐related programs, the present review does not focus on the receptor biochemistry itself. Instead, it emphasizes the tissue‐level roles of PDGFRβ‐positive stromal and perivascular cells in stem cell microenvironments [5, 6].

At the tissue level, PDGFRβ expression is enriched in pericytes, vascular smooth muscle‐like cells, and subsets of perivascular mesenchymal stromal cells, whereas most epithelial stem cell compartments appear to show a limited or indirect association with PDGFRβ expression [7]. This distribution distinguishes PDGFRβ from platelet‐derived growth factor receptor‐α (PDGFRα), which is more commonly associated with fibroblast‐like, interstitial, and connective tissue mesenchymal populations [5, 8, 9]. PDGFRβ‐positive perivascular cells have been linked to mural cell recruitment, vessel stabilization, vascular–stromal communication, extracellular matrix (ECM) organization, and tissue repair. In several organs, endothelial cell‐derived PDGF‐B supports the recruitment and maintenance of PDGFRβ‐expressing pericytes, thereby contributing to vascular integrity and perivascular niche architecture [10]. These observations suggest that PDGFRβ may influence stem cell behavior mainly through regulation of the surrounding niche rather than by acting as a direct stem cell‐intrinsic determinant [11–15].

The stem cell niche represents a dynamic regulatory unit composed of vascular elements, stromal constituents, ECM, immune components, and neural inputs. Its principal function is to maintain equilibrium among stem cell quiescence, self‐renewal, and differentiation [16, 17]. Within this framework, PDGFRβ‐positive stromal and perivascular cells may contribute to stem cell homeostasis through indirect microenvironmental mechanisms. First, by supporting vascular stability and local perfusion, these cells may affect oxygen availability, nutrient supply, and metabolic gradients, which are important extrinsic regulators of stem cell dormancy and activation [11, 18, 19]. Second, PDGFRβ‐associated stromal remodeling may alter ECM composition, tissue stiffness, and adhesion‐related signaling, thereby affecting mechanosensitive pathways such as YAP/TAZ and β‐catenin signaling [20–22]. Third, after injury or inflammation, increased PDGF ligand availability can activate PDGFRβ‐expressing stromal cells involved in vascular repair and matrix remodeling [23, 24]. Importantly, the biological consequence of PDGFRβ activation is likely to depend on its intensity, duration, and tissue context: transient activation may contribute to repair and niche restoration, whereas sustained or excessive activation may promote fibrosis, aberrant matrix deposition, and disruption of the stem cell‐supportive architecture [25–27].

These issues are particularly relevant to the LSC niche, which maintains corneal epithelial renewal through coordinated interactions among limbal epithelial stem/progenitor cells, stromal cells, nerves, immune cells, ECM, and vascular‐associated components. Disruption of this niche can lead to LSC deficiency (LSCD), a clinically important condition characterized by impaired corneal epithelial maintenance, conjunctivalization, chronic inflammation, neovascularization, and loss of corneal transparency. Although stromal remodeling, inflammation, and vascular changes are prominent features of many limbal disorders, the specific contribution of PDGFRβ‐positive stromal or perivascular cells to limbal niche maintenance remains insufficiently defined. In particular, direct receptor‐specific evidence for a functional role of PDGFRβ in the LSC microenvironment is still limited, and current hypotheses must therefore be interpreted cautiously in light of evidence from better‐characterized stem cell niches.

Accordingly, this review aims to clarify the potential role of PDGFRβ‐associated stromal and perivascular mechanisms in stem cell niche organization while distinguishing established evidence from tissue‐specific hypotheses. We first summarize cross‐tissue evidence linking PDGFRβ‐positive cells to vascular stability, stromal remodeling, ECM regulation, and injury responses in representative stem cell microenvironments. We then use this framework to discuss how similar mechanisms may be relevant to the LSC niche and LSCD. By explicitly defining this knowledge gap, the review provides a cautious cross‐tissue framework for future studies of PDGFRβ‐associated stromal and perivascular mechanisms in corneal and limbal biology.

## 3. Hematopoietic Stem Cell (HSC) Niche

### 3.1. Perivascular Organization of the HSC Niche

**Figure 2:**
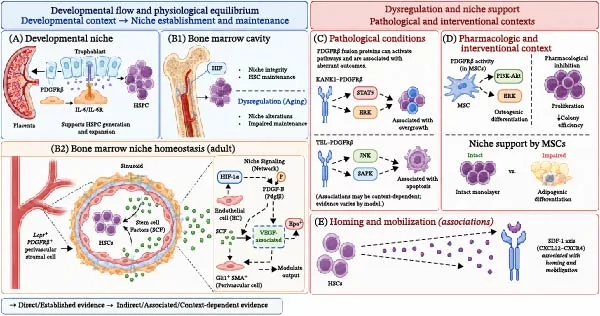
Context‐dependent PDGFRβ‐associated regulation of the hematopoietic stem cell niche. (A) In developmental hematopoiesis, placental PDGF‐B/PDGFRβ‐ and IL‐6/IL‐6R‐associated signaling supports hematopoietic stem and progenitor cell (HSPC) generation and expansion. (B1) In the adult bone marrow cavity, vascular–stromal organization contributes to niche integrity and hematopoietic stem cell (HSC) maintenance, whereas aging‐related dysregulation may impair niche support. (B2) In the adult bone marrow niche, Lepr^+^PDGFRβ^+^ perivascular stromal cells, endothelial cells, SCF‐associated support, HIF‐1α/PDGF‐B signaling, VEGF‐associated inputs, Gli1^+^SMA^+^ perivascular cells, and Epo‐related outputs collectively contribute to hematopoietic homeostasis. (C) Under pathological conditions, KANK1–PDGFRβ and TEL–PDGFRβ fusion proteins are associated with STAT5/ERK or JNK/SAPK pathway activation and context‐dependent hematopoietic dysregulation. (D) In pharmacologic and interventional contexts, PDGFRβ activity in MSCs supports PI3K–Akt and ERK signaling, proliferation, osteogenic differentiation, and niche‐supportive function, whereas pharmacological inhibition is associated with impaired stromal support and adipogenic bias. (E) HSC homing and mobilization are associated with the SDF‐1/CXCL12–CXCR4 axis and related microenvironmental cues. Solid arrows indicate direct or established evidence, whereas dashed arrows indicate indirect, associated, or context‐dependent relationships.

Stem cell niches sustain stem cell identity and long‐term functional competence through specialized supporting cellular components and soluble factors [15]. In the hematopoietic system, HSC maintenance depends on a spatially organized and functionally stratified bone marrow microenvironment. Genetic lineage tracing and three‐dimensional imaging analyses have consistently demonstrated that phenotypically defined HSCs preferentially localize to perisinusoidal regions. Crucially, the molecular signals required for HSC maintenance originate predominantly from perivascular stromal and endothelial populations rather than from hematopoietic cells themselves [11, 12, 28]. Leptin receptor‐positive (Lepr^+^) perivascular stromal cells exhibit spatial overlap with stem cell factor (SCF)‐expressing cells and display mesenchymal‐associated markers, including PDGFRβ, suggesting that PDGFRβ expression marks a subset of stromal elements within the perivascular HSC niche rather than defining a uniform niche cell population (Figure 2B1) [14].

### 3.2. Developmental and Homeostatic Regulation by PDGF‐B, Vascular Endothelial Growth Factor (VEGF), and HIF/Interleukin‐6 (IL‐6) Signaling

Within the placental hematopoietic microenvironment, endothelial‐derived PDGF‐B engages PDGFRβ expressed by trophoblast cells, limiting aberrant erythropoietin (Epo) upregulation and premature erythroid differentiation of hematopoietic stem and progenitor cells (HSPCs), thereby preserving a progenitor‐supportive niche state [29]. In parallel, VEGF has been reported, under nonhypoxic conditions, to induce Epo production in a subset of Gli1‐positive, smooth muscle actin‐positive (Gli1^+^SMA^+^) PDGFRβ^+^ perivascular stromal cells through a PDGFRβ‐associated mechanism. Through this context‐dependent mechanism, PDGFRβ^+^ niche cells may function as a conditional Epo reservoir, indirectly modulating erythroid lineage output from HSCs [30]. During embryonic hematopoietic development, stabilization of hypoxia‐inducible factor‐1α (HIF‐1α) upregulates PDGFβ and PDGFRβ expression and promotes HSPC generation and expansion through the IL‐6/IL‐6 receptor (IL‐6/IL‐6R) axis (Figure 2A) [31]. By contrast, aging or endothelial HIF dysregulation is associated with impaired PDGFRβ^+^ perivascular cell function and progressive deterioration of vascular niche integrity, ultimately compromising long‐term HSC support capacity [32]. Together, these findings suggest that PDGFRβ‐associated perivascular stromal elements can participate in the integration of PDGF‐B‐, VEGF‐, and HIF/IL‐6‐related signals in specific developmental or physiological contexts. Rather than indicating a single conserved pathway, the available evidence supports a context‐dependent role for PDGFRβ‐positive niche cells in regulating Epo output, vascular homeostasis, and stage‐specific HSC/HSPC production or differentiation (Figure 2B2).

### 3.3. Aberrant PDGFRβ Activation and Hematopoietic Disequilibrium

Under pathological conditions or sustained aberrant receptor activation, altered PDGFRβ signaling can disturb hematopoietic homeostasis. The KANK1–PDGFRβ fusion protein drives abnormal hematopoietic proliferation through activation of signal transducer and activator of transcription 5 (STAT5) and extracellular signal‐regulated kinase (ERK) pathways [33], whereas the TEL–PDGFRβ fusion protein induces apoptosis via the c‐Jun N‐terminal kinase/stress‐activated protein kinase (JNK/SAPK) cascade [34]. These findings support the view that constitutive PDGFRβ activation can perturb the hematopoietic lineage equilibrium (Figure 2C). HSC homing and mobilization are also shaped by microenvironmental molecular interactions, particularly the stromal cell‐derived factor‐1/C‐X‐C chemokine receptor type 4 (SDF‐1/CXCR4) axis. In this context, bone marrow‐derived HSCs may be mobilized through SDF‐1/CXCR4‐associated signaling and subsequently differentiate into endothelial progenitor cells that contribute to pathological neovascularization (Figure 1E) [35]. Because this process is mainly linked to the SDF‐1/CXCR4 axis, it should be interpreted as an example of microenvironmental control of HSC trafficking rather than direct evidence of PDGFRβ‐dependent regulation.

Within the bone marrow microenvironment, mesenchymal stem cells (MSCs) require PDGFRβ activity to sustain PI3K/protein kinase B (PI3K/Akt) and ERK signaling, thereby supporting proliferation and osteogenic differentiation. Preservation of an intact stromal monolayer architecture is essential for maintaining the long‐term expansion and clonogenic potential of CD34^+^ HSCs. Pharmacological inhibition of PDGFRβ reduces MSC proliferation and colony‐forming efficiency, enhances adipogenic differentiation, and markedly impairs their long‐term supportive capacity for CD34^+^ HSCs. These observations indicate that PDGFRβ indirectly governs HSC niche functionality by preserving MSC structural and biological integrity (Figure 2D) [36]. However, because these data are derived from stromal support models and pharmacological inhibition, they should be viewed as evidence for PDGFRβ‐dependent stromal contribution to HSC support rather than proof that PDGFRβ directly regulates HSC‐intrinsic stemness.

The evidence supporting PDGFRβ‐associated regulation in HSC niches is summarized in Table 1, with emphasis on experimental strength, functional relevance, and current limitations. Overall, evidence from the hematopoietic system supports a predominantly niche‐mediated role for PDGFRβ. Rather than functioning as an intrinsic determinant of HSC stemness, PDGFRβ is mainly associated with perivascular stromal cells and MSCs that provide structural and trophic support within the bone marrow niche. By coupling PDGF‐B‐, VEGF‐, and HIF/IL‐6‐related signals to vascular stability, metabolic and hypoxic cues, SCF‐ and Epo‐associated niche outputs, and MSC integrity, PDGFRβ may contribute to HSC/HSPC generation, lineage output, and long‐term maintenance. Conversely, persistent or aberrant PDGFRβ activation, including fusion protein‐mediated STAT5, ERK, or JNK signaling, can disrupt hematopoietic equilibrium and impair MSC‐mediated support. These findings illustrate a cross‐tissue principle in which PDGFRβ is best interpreted as a regulator of perivascular and stromal niche functions that can influence stem cell behavior indirectly rather than as a direct controller of stem cell identity (Figure 2).

**Table 1:** Evidence hierarchy of PDGFRβ‐associated regulation in hematopoietic stem cell niches.

Hematopoietic niche model	PDGFRβ‐associated cell population	Experimental model	Evidence category	Established function	Major limitation
Embryonic AGM hematopoietic niche [37]	PDGFRβ^+^ cells surrounding the dorsal aorta (AGM perivascular niche cells)	Pdgfrb genetic deletion, hematopoietic assays, transcriptomic profiling, lineage tracing	Direct genetic perturbation + lineage tracing (high‐confidence causal evidence)	PDGFRβ signaling is required for AGM niche activity and supports hematopoietic stem/progenitor cell generation during embryonic development	Evidence is mainly restricted to embryonic hematopoiesis; whether the same mechanism operates in adult HSC maintenance remains unclear
Placental hematopoietic niche [29]	PDGFRβ‐expressing placental niche cells	PDGF‐B/PDGFRβ pathway genetic disruption	Pathway‐level genetic manipulation (causal pathway evidence)	PDGF‐B/PDGFRβ signaling regulates placental hematopoietic microenvironment function by restricting inappropriate erythroid differentiation and maintaining developmental hematopoietic balance	Evidence represents a specialized developmental hematopoietic organ and may not directly translate to adult bone marrow niches
Bone marrow hematopoietic niche [38]	PDGFRβ‐expressing vascular‐associated niche cells	Spatial localization, cellular characterization, hematopoietic support studies	Spatial association + cellular characterization (associative evidence)	PDGFRβ expression identifies vascular‐associated cells located within HSC‐supportive regions of bone marrow and contributes to niche organization	Most evidence is based on cellular association; direct PDGFRβ‐specific manipulation in adult bone marrow remains limited

*Note:* Evidence is summarized by niche model, PDGFRβ‐associated cell population, experimental approach, functional implication, and major limitation.

## 4. MSC Niche

### 4.1. Perivascular Identity and Lineage Positioning of MSCs

Under steady‐state conditions across multiple tissues, MSCs predominantly localize to perivascular compartments, where they establish vascular‐associated stromal microenvironments through close interactions with endothelial cells and the ECM [39]. PDGFRβ, a commonly used marker of perivascular MSCs and pericyte‐like populations, contributes to MSC positioning along the vascular wall, thereby supporting niche organization and positional stability [40]. In organs, including the kidney [41], dental pulp [42], endometrium [43], and lung [44], PDGFRβ is enriched in perivascular and interstitial mesenchymal cell populations and has been associated with tissue architecture preservation, regenerative responses, and pathological remodeling. PDGFRβ expression also exhibits subpopulation specificity, enabling the discrimination of MSC subsets with distinct functional phenotypes and suggesting a role in stromal compartmentalization within local microenvironments [45]. During development, PDGFRβ is expressed in vascular‐associated progenitors undergoing mesenchymal lineage commitment and participates in the formation of MSCs and vascular support cells, supporting its involvement in the establishment of vascular‐associated mesenchymal lineages [46].

### 4.2. Endothelial–MSC Crosstalk in Vascular Assembly and Stabilization

Vessel formation and stabilization depend on coordinated crosstalk among MSCs, their perivascular derivatives, and endothelial cells. Endothelial‐derived PDGF‐B activates PDGFRβ signaling in MSCs, promoting directed migration toward vascular structures and supporting pericyte‐like differentiation or vascular‐supportive functions, thereby contributing to vascular wall assembly and maturation [39, 47, 48]. Concurrently, PDGFRβ^+^ MSCs can secrete angiogenic mediators such as VEGF and angiopoietin‐1 (Ang‐1), which enhance endothelial stability and vascular integrity through paracrine interactions [49, 50]. MSCs further recruit endogenous perivascular cells via PDGF–PDGFRβ signaling and promote their incorporation into nascent vascular networks, contributing to vascular maturation and long‐term functionality [39]. The evidence supporting PDGFRβ‐associated regulation in MSC microenvironments is summarized in Table 2, with emphasis on experimental strength, established function, and current limitations.

**Table 2:** Evidence hierarchy of PDGFRβ‐associated regulation in mesenchymal stem cell microenvironments.

MSC‐related microenvironment	PDGFRβ‐associated cell population	Experimental evidence	Evidence category	Established function	Major limitation
Skeletal MSC developmental niche [51]	PDGFRβ‐associated bone mesenchymal stromal cells (BMSCs)	Mouse genetic models, lineage tracing, single‐cell transcriptomic analysis, and differentiation assays	Genetic manipulation + lineage tracing	PDGFRβ signaling contributes to BMSC fate specification and osteogenic/stromal lineage differentiation during skeletal development	Evidence mainly derives from developmental bone models; whether this mechanism represents a general MSC niche regulation mechanism remains unclear
Adipose‐derived MSC niche [52]	CD140b (PDGFRβ)^+^ adipose‐derived MSCs	CD140b/PDGFRβ knockdown followed by proliferation, adhesion, migration assays and endothelial coculture experiments	Cell‐specific receptor knockdown	PDGFRβ contributes to intrinsic ASC properties, including proliferation, adhesion, migration, and angiogenic‐supportive activity	Findings are mainly obtained from isolated ASC models; in vivo niche‐level validation is limited
MSC migration and tissue repair niche [53]	PDGFRβ‐expressing MSCs responding to endothelial‐derived PDGF‐BB signals	PDGF‐BB stimulation, PDGFRβ knockdown/knockout, migration assays, and bone repair models	Receptor activation + functional perturbation	Endothelial‐derived PDGF‐BB/PDGFRβ signaling promotes MSC recruitment and migration during injury‐associated repair responses	Effects are mainly demonstrated in injury models and may represent regenerative responses rather than steady‐state niche maintenance
MSC extracellular matrix interaction niche [54]	PDGFRβ‐expressing MSCs responding to fibronectin/integrin‐mediated signals	Fibronectin stimulation, α5β1‐integrin manipulation, PDGFRβ phosphorylation analysis, and migration assays	ECM‐mediated receptor activation + pathway analysis	ECM signals activate PDGFRβ‐related pathways to regulate MSC migration and cytoskeletal responses	Demonstrates migratory regulation but does not directly establish long‐term MSC maintenance or stemness regulation
Perivascular MSC identity across tissues [55]	PDGFRβ^+^ mesenchymal/perivascular populations	Reporter analysis, immunostaining, single‐cell profiling, and spatial localization	Spatial association + marker expression	PDGFRβ identifies subsets of perivascular mesenchymal populations with MSC‐like characteristics	PDGFRβ expression indicates cellular identity or association but does not alone demonstrate functional dependence

*Note:* Evidence is summarized by MSC‐related microenvironment, PDGFRβ‐associated cell population, experimental approach, established function, and major limitation.

### 4.3. ECM‐Dependent Remodeling and Fibrotic Conversion of PDGFRβ^+^ Stromal Cells

As a central component of the MSC niche, the ECM functions as a critical determinant of MSC behavior and tissue repair outcomes. Fibronectin, an ECM constituent, induces ligand‐independent phosphorylation of PDGFRβ through α5β1 integrin engagement, leading to the activation of PI3K/Akt signaling and cytoskeletal reorganization. This mechanism enhances MSC motility and recruitment to sites of vascular remodeling and tissue injury [56]. PDGFRβ^+^ mesenchymal populations represent an important source of ECM production and, under injury conditions, may transition into myofibroblasts characterized by the expression of α‐smooth muscle actin (αSMA), collagen type I alpha 1 chain (Col1a1), and matrix metalloproteinases (MMPs), thereby contributing to collagen deposition and fibrotic matrix accumulation. Moreover, PDGFRβ^+^ cells have been implicated in the local activation of transforming growth factor‐β (TGF‐β) via αv integrins, promoting MSC differentiation toward an ECM‐producing phenotype and sustaining fibrotic microenvironments. Disruption of this signaling cascade attenuates TGF‐β activation and reduces ECM deposition, thereby mitigating structural deterioration. These findings indicate that PDGFRβ may participate in fibrotic niche remodeling through coordinated ECM–integrin–TGF‐β signaling interactions [46, 57].

### 4.4. Adhesion, Tissue Repair, and Regenerative Function in the MSC Niche

PDGFRβ also influences the MSC adhesive properties. N‐cadherin‐mediated cell–cell adhesion activates the PDGFRβ–Src signaling axis and induces expression of vascular cell adhesion molecule‐1 (VCAM‐1), strengthening interactions between MSCs and adjacent cellular or matrix components and potentially contributing to stable residence within perivascular domains [58]. In addition, PDGFRβ signaling sustains MSC proliferative capacity and stem‐like properties, supporting long‐term self‐renewal and niche integrity [59]. During tissue repair, PDGFRβ functions as an important mediator in MSCs and MSC‐like pericytes, contributing to the reconstruction of injury‐associated microenvironments [53, 54]. PDGFRβ^+^ MSCs display enhanced survival and engraftment potential and promote angiogenesis and tissue regeneration through the secretion of proangiogenic factors [50]. In addition, PDGFRβ^+^ pericytes can differentiate into osteoblasts and directly participate in bone regeneration [60, 61]. MSC‐derived extracellular vesicles further influence macrophage polarization and activate PDGF–PDGFRβ‐associated pathways, indirectly improving the inflammatory milieu and facilitating tissue repair [62]. However, these regenerative effects are context‐dependent and should not be interpreted as evidence that PDGFRβ universally determines MSC stemness across all tissues.

Collectively, PDGFRβ acts as a vascular–stromal regulatory component in the MSC niche rather than as a direct intrinsic determinant of MSC stemness. By integrating vascular‐derived growth factor signals, ECM‐associated adhesion cues, and injury‐induced stimuli, PDGFRβ may coordinate vascular support, matrix remodeling, and biomechanical signaling, thereby influencing MSC survival, migration, lineage specification, and regenerative potential across homeostatic, reparative, and pathological contexts. Current evidence is strongest for PDGFRβ‐associated regulation of perivascular positioning, stromal remodeling, and repair responses, whereas its direct and universal role in MSC fate determination remains tissue‐ and model‐dependent.

## 5. Neural Stem Cell (NSC) Niche

### 5.1. Developmental and Adult Organization of the NSC Niche

NSCs are sustained within highly specialized microenvironmental architectures collectively referred to as the NSC niche [63]. During embryonic development, this niche is primarily localized to the ventricular zone (VZ) and subventricular zone (SVZ), whereas in the adult brain, it persists within the ventricular–SVZ (V‐SVZ) and the subgranular zone (SGZ) of the dentate gyrus (DG) [64]. The NSC niche comprises a multilayered structural and signaling network including vascular components, perivascular cells, radial glia (RG), astrocytes, ECM, and locally derived inflammatory and metabolic cues. Together, these elements support NSC self‐renewal, multilineage differentiation, and neural tissue homeostasis [65, 66].

Within this hierarchical framework, PDGFRβ provides a potential structural and signaling link between the vascular architecture and progenitor cell regulation [67, 68]. PDGFRβ is predominantly expressed in pericytes and, during development, in subsets of RG cells, positioning it at the interface between vascular scaffolding and neural progenitor dynamics. In the developing human neocortex, PDGF‐D/PDGFRβ signaling regulates RG cell cycle progression. Inhibition of this pathway disrupts RG expansion and compromises germinal zone integrity, thereby influencing cortical structural development [69]. In the adult NSC niche, PDGFRβ^+^ pericytes envelop capillaries to form a perivascular framework that spatially associates with Nestin^+^ NSCs and doublecortin‐positive (DCX^+^) immature neurons. This anatomical proximity supports a spatial association between vascular positioning and stem cell activity [70, 71]. Following ischemic or traumatic injury, PDGFRβ^+^ cells colocalize with newly generated neural progenitors along the vascular basement membrane and may acquire Nestin expression, suggesting injury‐induced phenotypic plasticity and possible participation in regenerative niche reconstruction [70]. Beyond vascular positioning, PDGFRβ has been associated with niche organization by influencing ECM deposition and matrix remodeling, thereby shaping the spatial environment governing progenitor anchorage and migration [72]. Matrix mechanical properties further affect NSC fate via CD44‐mediated PDGFRβ signaling, indicating that this receptor axis may contribute to the transduction of biomechanical inputs into alterations in stem cell multipotency [73]. Conversely, pericyte depletion or PDGFRβ dysfunction leads to vascular barrier compromise and persistent inflammatory responses, which may secondarily destabilize the perivascular niche equilibrium [74].

### 5.2. Growth Factor Integration and Neural Progenitor Regulation

At the cellular level, loss of PDGFRβ attenuates neural stem and progenitor cell (NSPC) proliferation, self‐renewal capacity, and neurosphere formation efficiency, implicating this receptor in the maintenance of stem‐like properties in specific experimental models [75, 76]. PDGFRβ signaling interfaces with essential trophic factors, including brain‐derived neurotrophic factor (BDNF), fibroblast growth factor 2 (FGF2), and noggin. Reduced expression of these growth factors under PDGFRβ deficiency, together with partial rescue following exogenous supplementation, indicates that PDGFRβ may act as a context‐dependent signaling component linking vascular support to growth factor networks [75]. Furthermore, platelet‐derived growth factor‐BB (PDGF‐BB)/PDGFRβ signaling promotes neural progenitor proliferation and neuronal differentiation and cooperates functionally with ephrin type‐A receptor 4 (EphA4), placing PDGFRβ within a broader receptor interaction network [77]. In the DG, activation of the PDGF‐BB/PDGFRβ axis enhances adult neurogenesis and modulates behavioral outputs, whereas NSC‐specific PDGFRβ knockdown reduces neurogenic capacity and induces depressive‐like phenotypes, suggesting a possible link between PDGFRβ‐dependent neurogenesis and neural circuit‐related outcomes [78]. The evidence supporting PDGFRβ‐associated regulation in NSC niches is summarized in Table 3, with emphasis on the experimental context, evidence strength, established function, and current limitations.

**Table 3:** Evidence hierarchy of PDGFRβ‐associated regulation in neural stem cell microenvironments.

NSC‐related microenvironment	PDGFRβ‐associated cell population	Experimental evidence	Evidence category	Established function	Major limitation
Adult hippocampal neurogenic niche [78]	Hippocampal neural stem/progenitor cells	PDGF‐BB stimulation or depletion; NSC‐specific PDGFRβ knockdown; analysis of proliferation and neurogenesis	Ligand activation + cell‐specific receptor knockdown	PDGF‐BB/PDGFRβ signaling promotes NSC proliferation and supports adult neurogenesis under stress‐related conditions	Evidence is mainly derived from pathological stress models; physiological NSC maintenance requires further validation
Adult ventricular–subventricular zone (V‐SVZ) niche [76]	PDGFRβ‐expressing neural stem/progenitor cells	PDGFRβ genetic deletion; NSC self‐renewal and differentiation assays	Genetic loss‐of‐function	PDGFRβ contributes to NSC maintenance, proliferation, and fate regulation	Direct evidence is limited to specific developmental/adult niche models
Neural injury‐induced regenerative niche [71]	PDGFRβ‐associated neural progenitor‐associated environment	PDGFR inhibitor treatment or PDGFRβ pathway modulation in injury models; analysis of neurogenic responses and tissue remodeling	Receptor inhibition/pathway perturbation	PDGFRβ signaling participates in injury‐associated neural regenerative responses	Pharmacological or pathway‐level perturbation cannot establish direct NSC‐specific regulation
Neural stem cell migratory niche [79]	Neural stem/progenitor cells expressing PDGFRβ‐related signaling components	PDGFRβ expression analysis combined with migration assays	Spatial association + marker evidence	PDGFRβ expression is associated with neural progenitor migratory properties	No direct PDGFRβ‐specific functional manipulation

*Note:* Evidence is summarized by neural niche context, PDGFRβ‐associated cell population, experimental evidence, functional implication, and major limitation.

### 5.3. Injury‐Induced Remodeling and Regenerative Plasticity

Under pathological conditions, PDGFRβ contributes to injury‐associated niche remodeling. Following stroke, PDGFRβ enhances neuroblast migration within the ischemic ECM through regulation of C‐X‐C motif chemokine ligand 12 (CXCL12) and integrin expression, consistent with a possible chemotaxis–adhesion–migration mechanism [71]. In ischemia–reperfusion models, PDGFRβ^+^ pericytes may undergo phenotypic transition toward induced NSC‐like states, contributing to the reconstruction of an injury‐associated perivascular microenvironment [70]. In contrast, during severe injuries such as spinal cord trauma, PDGFRβ^+^ cells participate in scar boundary formation and mesenchymal proliferation [80]. Pharmacological inhibition in this context attenuates regenerative barriers, suggesting context‐ and stage‐dependent roles in regeneration and fibrotic containment [81].

### 5.4. Engineering Modulation and Pathological Extension of PDGFRβ Signaling

Engineering strategies further underscore the potential for therapeutic modulation of this pathway. PDGF‐mimetic hydrogel microspheres that enhance PDGFRβ activation improve NSC survival, differentiation, and functional restoration in injured environments, suggesting that receptor activation efficiency may represent a tunable regenerative parameter [82]. In vitro analyses indicate that PDGFRβ‐associated signaling regulates NSC migration and structural adaptation to the perivascular architecture, providing mechanistic insight into niche‐guided motility [79, 83]. In tumorigenic NSC niches, PDGFRβ signaling has been reported to support glioblastoma and medulloblastoma stem cell survival and self‐renewal, and pathway inhibition compromises microenvironmental support, extending the relevance of PDGFRβ from physiological neurogenesis to pathological stem cell ecosystems [84–86].

In the neural niche, PDGFRβ appears to operate at multiple levels rather than through a single regulatory mode. It can affect neural progenitor proliferation and differentiation in selected developmental, adult, or injury‐related settings while also shaping the surrounding perivascular, matrix, mechanical, and trophic environments. This dual pattern distinguishes the neural system from niches in which PDGFRβ acts mainly through stromal support and highlights the need to interpret its function according to the developmental stage, injury status, experimental model, and pathological context. Current evidence therefore supports a context‐dependent role for PDGFRβ in NSC regulation, while caution is required when extrapolating these findings to other stem cell niches, including the limbal microenvironment.

## 6. LSC Niche

### 6.1. Anatomical Organization and Established PDGFRβ‐Related Evidence in the Limbal Niche

**Figure 3:**
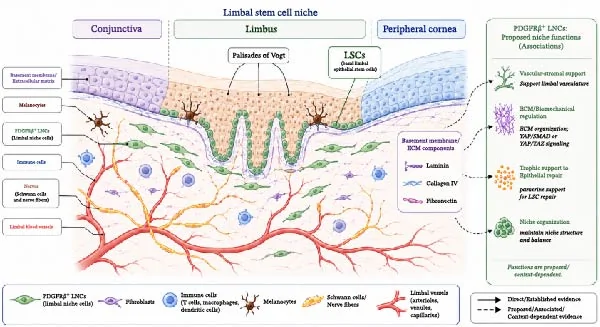
Proposed model of PDGFRβ^+^ LNCs in the limbal stem cell niche. The schematic shows PDGFRβ^+^ limbal niche cells (LNCs) in relation to the Palisades of Vogt, limbal epithelial stem/progenitor cells, vessels, nerves, melanocytes, immune cells, and basement membrane/ECM. Solid arrows indicate established niche relationships, whereas dashed arrows indicate proposed PDGFRβ‐associated functions in stromal support, ECM/biomechanical regulation, vascular–stromal organization, and epithelial repair.

The LSC niche is located at the corneoscleral limbus, where limbal epithelial stem/progenitor cells reside within specialized anatomical structures, including the Palisades of Vogt and limbal epithelial crypt‐like regions [87, 88]. This niche comprises basement membrane and ECM, limbal niche cells (LNCs), vascular cells, immune cells, melanocytes, Schwann cells, and soluble factors, which together support epithelial stem cell maintenance, regeneration, and wound‐responsive remodeling [89–92]. Single‐cell and lineage‐tracing studies further indicate that the limbal epithelium contains spatially distinct LSC populations, with outer limbal LSCs contributing to wound healing and boundary maintenance and inner limbal LSCs supporting epithelial homeostasis [93]. Melanocytes contribute to epithelial progenitor maintenance, immune regulation, and angiostasis, whereas limbal Schwann cells are required for innervation‐dependent corneal epithelial renewal [90, 91]. These features define the limbus as a specialized epithelial stem cell niche rather than a passive boundary between the cornea and conjunctiva [87, 88, 93]. These anatomical relationships and the proposed PDGFRβ‐associated stromal/perivascular functions in the LSC niche are schematically summarized in Figure 3.

Within this niche, PDGFRβ is predominantly associated with LNCs rather than limbal epithelial stem cells themselves. LNCs around the Palisades of Vogt display mesenchymal and perivascular‐like features, including expression of CD73, CD90, CD105, PDGFRβ, and αSMA, while remaining distinct from hematopoietic and epithelial populations [94]. Gene expression profiling further shows that LNCs differ from bone marrow‐derived MSCs, with enrichment of ECM–receptor interaction and Wnt signaling pathways accompanied by elevated PDGFRβ expression [95]. LNCs also exhibit tissue‐specific stromal progenitor features, including neural crest‐associated markers and SCF‐related niche‐supportive potential [96].

Taken together, current limbal evidence supports PDGFRβ expression, stromal niche cell identity, and spatial association within the limbal microenvironment. However, these observations do not demonstrate that PDGFRβ signaling directly regulates the LSC fate, vascular stability, or ECM remodeling. Therefore, PDGFRβ should be described as an LNC‐associated stromal/perivascular marker and candidate regulatory molecule in the limbal niche, whereas its functional contribution requires validation through targeted perturbation studies. The current evidence and remaining limitations of PDGFRβ‐associated regulation in the LSC niche are summarized in Table 4.

**Table 4:** Evidence hierarchy of PDGFRβ‐associated features in limbal stem cell microenvironments.

LSC‐related microenvironment	PDGFRβ‐associated cell population	Experimental evidence	Evidence category	Established function	Major limitation
Human limbal epithelial stem cell niche [95]	LNCs surrounding limbal epithelial progenitors	Isolation of human LNCs; transcriptomic analysis; qPCR, western blot and immunostaining of niche‐associated markers including PDGFRβ	Spatial association + marker expression	PDGFRβ is a characteristic marker of Limbal niche cells associated with epithelial stem/progenitor cell‐supportive properties	No PDGFRβ‐specific manipulation; direct regulatory function remains unknown
Limbal stromal niche characterization [97]	PDGFRβ‐expressing limbal niche cells	Molecular characterization of human/rabbit LNCs using immunostaining, gene expression analysis and protein detection	Marker expression across species	PDGFRβ contributes to identification of limbal stromal niche populations	Expression indicates cellular identity but does not demonstrate PDGFRβ‐dependent regulation
LNC‐mediated limbal epithelial progenitor support [98]	PDGFRβ‐positive limbal niche stromal cells	LNC–corneal epithelial cell coculture; 3D culture systems; analysis of epithelial progenitor markers	Functional niche association	LNCs support epithelial progenitor‐like characteristics and maintenance of limbal epithelial phenotype	The functional effect represents the entire LNC population and cannot be attributed specifically to PDGFRβ
Disease‐associated limbal niche dysfunction [99]	Human LNCs with altered PDGFRβ expression under pathological stress	Disease/stress models; expression analysis of PDGFRβ and niche functional markers	Disease‐associated marker alteration	PDGFRβ expression changes accompany limbal niche dysfunction	PDGFRβ alteration may be secondary to niche damage rather than a causal driver

*Note:* Evidence is summarized by limbal niche component, PDGFRβ‐associated feature, experimental basis, proposed relevance, and major limitation.

### 6.2. Vascular–Stromal and ECM‐Associated Mechanisms: From Established Niche Features to Proposed PDGFRβ Functions

The limbus contains blood and lymphatic vascular elements that are closely integrated with stromal niche cells, immune components, melanocytes, nerves, and the basement membrane surrounding epithelial progenitors [89–91]. Vascular endothelial cells are recognized components of the LSC niche and may contribute to nutrient supply, inflammatory regulation, Wnt‐related signaling, and epithelial repair responses [92]. ATP‐binding cassette subfamily B member 5‐positive (ABCB5^+^) limbal epithelial stem cells and limbal melanocytes can regulate blood or lymphatic vascular behavior in a context‐dependent manner, indicating that vascular regulation is an active feature of the limbal niche [100].

Because PDGFRβ is a well‐established marker and regulator of perivascular stromal cells in other tissues, PDGFRβ^+^ LNCs or perivascular stromal cells, are plausible candidate contributors to vascular–stromal organization around the Palisades of Vogt. In the limbus, however, this remains an inferred mechanism rather than direct evidence. Limbal‐specific PDGFRβ inhibition, knockdown, or gain‐of‐function experiments are still required to determine whether PDGFRβ signaling controls vascular stability or vascular–stromal communication. Accordingly, the following mechanisms should be interpreted as hypothesis‐generating links between established limbal niche biology and cross‐tissue PDGFRβ evidence rather than as confirmed limbal‐specific PDGFRβ functions.

ECM and basement membrane organization provide another relevant link between PDGFRβ^+^ LNCs and epithelial progenitor regulation. The limbal ECM supports cell adhesion, migration, proliferation, self‐renewal, and differentiation, while basement membrane heterogeneity contributes to the distinct microenvironment of epithelial progenitors [101, 102]. The human limbal basement membrane is enriched for laminin α2 and α5 chains, and laminin‐511 and laminin‐521 matrices enhance limbal epithelial progenitor adhesion, migration, proliferation, and phenotype stability [102]. Biomechanical studies further show that limbal stiffness supports YAP‐associated stem cell maintenance, whereas altered stiffness or YAP/SMAD signaling affects both LSC function and the LNC phenotype [103, 104]. These data establish ECM composition and mechanical properties as functional regulators of the limbal niche, but the specific role of PDGFRβ in limbal ECM remodeling remains unresolved.

LNCs support limbal epithelial progenitors through direct contact, paracrine signaling, extracellular vesicles, and matrix‐associated cues. SDF‐1/CXCR4 signaling mediates interactions between limbal epithelial stem/progenitor cells and stromal niche cells, bone morphogenetic protein (BMP)/Wnt signaling regulates clonal growth, and three‐dimensional Matrigel coculture with LNCs can induce mature corneal epithelial cells toward a less differentiated, progenitor‐like state [98, 105, 106]. In vivo and ex vivo studies show that LNCs or limbal stromal stem/progenitor cells improve epithelial repair, reduce neovascularization and goblet cell density, support collagen‐rich ECM reconstruction, and provide antiangiogenic or anti‐inflammatory niche factors [96, 107]. LNC‐derived exosomes also promote corneal epithelial proliferation and migration through PI3K/Akt activation and forkhead box O3 (FOXO3) inhibition [108]. These studies demonstrate the functional relevance of LNC‐mediated niche support, but they do not establish whether PDGFRβ signaling is required for these effects.

### 6.3. Pathological Relevance, Knowledge Gaps, and Future Experimental Directions

Pathological disruption of the limbal niche involves epithelial progenitor dysfunction together with stromal, vascular, inflammatory, neural, and ECM‐related changes. LSCD is characterized by corneal conjunctivalization, neovascularization, opacity, chronic inflammation, scarring, and visual loss, reflecting combined epithelial and niche failure [89, 92, 109]. In diabetic or hyperglycemic conditions, LNC dysfunction is accompanied by reduced proliferation, increased oxidative stress and senescence, and decreased expression of SCF, PDGFRβ, and paired box 6 (PAX6); diabetic limbal tissue also shows reduced ATP‐binding cassette subfamily G member 2 (ABCG2), p63, and PAX6 expression [99]. Exosomes from normal corneolimbal keratocytes promote limbal epithelial cell proliferation, migration, Akt activation, and keratin 15 (K15)/Frizzled‐7 expression more effectively than diabetic keratocyte‐derived exosomes, indicating that metabolic disease can impair stromal paracrine support [110]. These findings indicate that reduced PDGFRβ expression may accompany limbal niche destabilization, but whether it is causal remains undetermined.

Future studies should directly test PDGFRβ function in LNCs using inhibition, knockdown, or overexpression. Spatial mapping should define the relationship among PDGFRβ^+^ LNCs, CD31^+^ limbal vessels, melanocytes, Schwann cells, immune cells, basement membrane components such as laminin, collagen IV, and fibronectin, and p63α^+^/ABCG2^+^/K15^+^ epithelial progenitors [90–92, 102]. Coculture, exosome‐based, and organoid‐like models could assess whether PDGFRβ‐modified LNCs affect colony‐forming efficiency, epithelial migration, wound closure, keratin 3/keratin 12 (K3/K12) expression, loss or restoration of K12, ECM remodeling, PI3K/Akt activity, FOXO3 inhibition, and YAP/TAZ‐ or YAP/SMAD‐associated mechanotransduction [104, 108]. Controlled LSCD and epithelial injury models, including mechanical, thermal, alkali burn, diabetic, and epithelial defect models, provide relevant in vivo readouts such as corneal epithelial defects, fluorescein staining, superficial neovascularization, goblet cell appearance, corneal opacity, and stromal scarring. These approaches would help distinguish whether PDGFRβ is functionally required in LNCs or instead serves mainly as a marker of the stromal niche state.

Overall, the limbal niche suggests a context in which PDGFRβ may contribute to LNC‐associated stromal support rather than directly regulating the LSC identity. Current evidence links PDGFRβ^+^ LNCs to limbal stromal identity and niche organization, while their roles in vascular stability, ECM remodeling, and epithelial repair remain largely inferential. Direct PDGFRβ perturbation in LNCs will therefore be required to define its functional relevance in LSC maintenance and LSCD repair. Thus, PDGFRβ should currently be framed as a plausible but insufficiently validated component of limbal stromal/perivascular regulation rather than as an established driver of LSC fate or LSCD recovery.

## 7. Discussion

Accumulating evidence across multiple tissues indicates that PDGFRβ is frequently associated with vascular–stromal organization within stem cell niches [111, 112]. Across diverse biological systems, PDGFRβ signaling is predominantly associated with perivascular and mesenchymal stromal populations rather than with stem cells themselves [113]. This cellular distribution is consistent with a niche‐mediated model in which PDGFRβ may influence stem cell behavior indirectly by regulating vascular organization, stromal activity, ECM dynamics, and paracrine signaling rather than by directly controlling stem cell‐intrinsic transcriptional programs [114, 115]. However, this model must be interpreted according to evidence strength: receptor‐specific functional evidence is stronger in selected hematopoietic, mesenchymal, and neural models, whereas evidence in the limbal niche remains largely limited to PDGFRβ expression, stromal identity, and spatial association.

The studies summarized in this review suggest that PDGFRβ functions not as a universal determinant of stemness but as a context‐dependent vascular–stromal regulatory component [116, 117]. By coordinating interactions among endothelial cells, pericytes, and stromal progenitors, PDGFRβ can contribute to tissue environments that support stem cell residence and responsiveness [118, 119]. Rather than directly controlling lineage specification, PDGFRβ appears to regulate the permissive conditions under which stem cells transition among quiescence, activation, and differentiation [112, 114]. Similar PDGFRβ‐associated vascular–stromal features recur across tissues, but their functional consequences differ according to the identity and position of PDGFRβ‐expressing cells, the relevant PDGF ligands, and the experimental strength of the available evidence [37, 120].

A related feature emerging from current evidence is the coupling between the physical niche organization and biochemical signaling networks [114, 116]. Stromal elements expressing PDGFRβ simultaneously influence cellular positioning, ECM composition, and paracrine factor availability. These components collectively shape the microenvironmental landscape to which stem cells are exposed [113, 121]. Accordingly, perturbation of PDGFRβ signaling may alter stem cell behavior by reshaping the surrounding niche architecture rather than by directly reprogramming intrinsic stem cell fate. This distinction is essential because marker expression, spatial colocalization, and receptor‐specific perturbation represent different evidence levels. Only receptor‐specific inhibition, deletion, knockdown, or gain‐of‐function studies can establish a direct functional role for PDGFRβ.

The apparently divergent outcomes of PDGFRβ‐associated signaling can be explained by a context–duration–threshold model. In physiological or reparative settings, transient and spatially restricted PDGF–PDGFRβ activation may support pericyte recruitment, vessel stabilization, stromal migration, controlled ECM deposition, and paracrine repair signals, thereby contributing to niche restoration. In contrast, persistent, excessive, or spatially disorganized activation can convert similar processes into maladaptive remodeling, including myofibroblast expansion, excessive collagen deposition, pathological vascular remodeling, chronic inflammatory activation, or tumor‐supportive perivascular niches. Thus, the same PDGFRβ‐associated programs may support repair when activation is limited and organized but may promote fibrosis, pathological angiogenesis, or tumor progression when activation is sustained, dysregulated, or embedded in inflammatory or malignant microenvironments. This interpretation provides a more coherent basis for the cross‐tissue PDGFRβ framework.

Despite growing recognition of its role in niche regulation, several questions remain unresolved [122]. A major issue is not only how PDGFRβ signaling intersects with canonical niche pathways, including Wnt, Notch, and chemokine signaling systems [123] but also whether, in a given tissue context, PDGFRβ functions as an upstream or permissive niche‐level integrator coupling vascular–stromal organization to stem cell maintenance. A cross‐tissue comparative perspective may help clarify whether PDGFRβ functions as a convergent regulatory node within these networks or instead operates through context‐dependent mechanisms [124]. In addition, the temporal regulation of PDGFRβ during development, aging, tissue repair, and pathological remodeling remains insufficiently characterized [125]. Future work should therefore define not only the presence of PDGFRβ^+^ niche cells but also the timing, intensity, and cellular source of PDGF–PDGFRβ signaling during homeostasis, injury, and repair [126]. Notably, recent preclinical studies in the tumor stem cell niche further suggest that PDGFRβ‐targeted intervention may disrupt stromal recruitment, perivascular support, and therapy‐resistant niche maintenance, thereby providing a rationale for combination treatment strategies [127–129]. However, tumor‐associated findings should not be directly extrapolated to normal epithelial stem cell niches because tumor microenvironments involve persistent inflammatory, angiogenic, and stromal activation states. In contrast, therapeutic implications in the limbal niche remain more preliminary because direct PDGFRβ‐specific functional evidence in LNCs is still limited. Any future microenvironment‐targeted strategy in the cornea will therefore require careful validation of cell specificity, timing, and effects on vascular stability, stromal support, and epithelial regeneration. Because PDGFRβ is also involved in fundamental processes such as vascular stability and stromal organization, therapeutic manipulation will require precise spatial and temporal control [130].

In this context, the corneal LSC niche may provide a particularly informative model for addressing the unresolved relationship between PDGFRβ‐associated stromal regulation and epithelial stem cell maintenance [131]. The corneal epithelium is maintained by LSCs located at the Palisades of Vogt, where epithelial progenitors interact closely with stromal niche cells, ECM components, and vascular structures [87]. Increasing evidence suggests that limbal stromal cells share phenotypic characteristics with mesenchymal niche populations identified in other tissues, including the expression of markers associated with perivascular support cells [94, 132]. Within the limbal niche, current evidence mainly establishes PDGFRβ expression in LNCs, their stromal/perivascular identity, and their spatial association with epithelial progenitors, vessels, and ECM components. By contrast, PDGFRβ‐specific roles in vascular stability, ECM remodeling, biomechanical signaling, and LSC‐supportive function remain proposed mechanisms. Given the established role of PDGFRβ in stromal–vascular interactions in other tissues, a testable model is that PDGFRβ signaling in LNCs may contribute indirectly to the microenvironment required for LSC homeostasis [133]. This should be interpreted as a cross‐tissue hypothesis rather than evidence for PDGFRβ‐dependent limbal microenvironmental regulation. This question is experimentally tractable in the limbal niche, where niche architecture, stromal cell populations, and epithelial regenerative outcomes can be examined in parallel [134]. In particular, determining whether disruption of PDGFRβ signaling impairs stromal support, vascular–stromal communication, and ECM remodeling in the limbal region could improve understanding of stem cell dysfunction in corneal diseases such as LSCD [135, 136]. Such work may help define whether PDGFRβ is functionally relevant to limbal niche dysfunction and repair rather than assuming a causal role from marker expression alone [137]. Until receptor‐specific perturbation studies are performed in LNCs or limbal stromal/perivascular cells, PDGFRβ should be described as a candidate regulator or marker‐associated pathway in the limbal niche, not as a proven driver of LSC maintenance or LSCD repair.

The dedicated limbal model figure further summarizes this evidence hierarchy by separating established anatomical relationships from proposed PDGFRβ‐associated functions in stromal support, ECM/biomechanical regulation, vascular–stromal organization, and epithelial repair (Figure 2) [87, 97]. Future studies should therefore prioritize spatial mapping of PDGFRβ^+^ LNCs relative to the Palisades of Vogt, limbal vessels, basement membrane/ECM components, and LSCs, together with PDGFRβ‐specific inhibition, knockdown, or overexpression in LNC‐based coculture, organoid‐like, and in vivo LSCD models [96, 102]. These experimental directions directly address whether PDGFRβ functions as an active regulator of limbal niche stability or primarily serves as a marker of specialized stromal/perivascular niche identity [108].

Overall, the evidence summarized in this review supports a model in which PDGFRβ is associated with context‐dependent vascular–stromal regulation in stem cell niches. By influencing structural and signaling components of the microenvironment, PDGFRβ may indirectly affect stem cell behavior across multiple tissues. In this framework, the LSC niche represents a clinically relevant system for testing whether PDGFRβ‐associated stromal signaling links the vascular–stromal architecture to epithelial stem cell maintenance and repair [138]. At present, however, the limbal component of this framework remains hypothesis‐generating, and causal terms such as “PDGFRβ‐dependent limbal microenvironmental regulation” should be reserved for future studies demonstrating receptor‐specific functional effects in LNCs.

## 8. Conclusion

The collective evidence reviewed here highlights PDGFRβ as a recurrent vascular–stromal component associated with stem cell niche organization across diverse biological systems. Although stem cells themselves often exhibit limited PDGFRβ expression, neighboring stromal and perivascular cells enriched in this receptor may help shape the microenvironment in which stem cells reside. Across hematopoietic, mesenchymal, neural, tumor, renal glomerular, and limbal niches, PDGFRβ most commonly acts through vascular–stromal organization, ECM remodeling, paracrine signaling, and biomechanical regulation rather than through direct control of stem cell identity. This context‐dependent mode of action supports the interpretation of PDGFRβ as a niche‐associated vascular–stromal regulatory component rather than a universal or directly causal determinant of stem cell fate.

For the LSC niche, current evidence mainly establishes PDGFRβ expression in LNCs, their stromal/perivascular identity, and their spatial association with the Palisades of Vogt, epithelial progenitors, limbal vessels, and basement membrane/ECM. However, whether PDGFRβ signaling directly contributes to LNC‐mediated vascular support, ECM remodeling, biomechanical regulation, epithelial repair, or LSCD recovery remains unresolved. Future studies using spatial mapping, PDGFRβ‐specific perturbation in LNCs, coculture or organoid‐like assays, and in vivo LSCD models will be required to define its functional role in limbal niche stability and corneal epithelial regeneration. Therefore, the limbal component of this review should currently be viewed as a hypothesis‐generating framework derived from cross‐tissue evidence, not as established evidence that PDGFRβ directly regulates the limbal microenvironment. Taken together, the present review emphasizes PDGFRβ‐associated vascular–stromal mechanisms across tissues while highlighting their potential, but still insufficiently validated, relevance to the LSC niche.

## Author Contributions

All authors contributed to researching the data for the article and writing the article. Shuying Liao contributed to writing – original draft preparation. Guigang Li contributed to writing – review and discussion.

## Funding

This research was funded and supported by the National Natural Science Foundation of China (Grants 82471050 and 82070936).

## Disclosure

All authors read and approved the final version of the work to be published.

## Conflicts of Interest

The authors declare no conflicts of interest.

## Data Availability

Data sharing is not applicable to this article as no datasets were generated or analyzed during the current study.

## References

[1] Lemmon M. A. and Schlessinger J. , Cell Signaling by Receptor Tyrosine Kinases, Cell. (2010) 141, no. 7, 1117–1134, 10.1016/j.cell.2010.06.011. PMC2914105 20602996

[2] Yarden Y. , Escobedo J. A. , and Kuang W.-J. , et al.Structure of the Receptor for Platelet-Derived Growth Factor Helps Define a Family of Closely Related Growth Factor Receptors, Nature. (1986) 323, no. 6085, 226–232, 10.1038/323226a0. 3020426

[3] Roskoski R.Jr., The Role of Small Molecule Platelet-Derived Growth Factor Receptor (PDGFR) Inhibitors in the Treatment of Neoplastic Disorders, Pharmacological Research. (2018) 129, 65–83, 10.1016/j.phrs.2018.01.021. 29408302

[4] Wang F. , Ye Y. , and Zhang Z. , et al.PDGFR in PDGF-BB/PDGFR Signaling Pathway Does Orchestrates Osteogenesis in a Temporal Manner, Research. (2023) 6, 10.34133/research.0086, 0086. PMC10202377 37223474

[5] Andrae J. , Gallini R. , and Betsholtz C. , Role of Platelet-Derived Growth Factors in Physiology and Medicine, Genes & Development. (2008) 22, no. 10, 1276–1312, 10.1101/gad.1653708. PMC2732412 18483217

[6] Heldin C.-H. and Westermark B. , Mechanism of Action and In Vivo Role of Platelet-Derived Growth Factor, Physiological Reviews. (1999) 79, no. 4, 1283–1316, 10.1152/physrev.1999.79.4.1283. 10508235

[7] Armulik A. , Genové G. , and Betsholtz C. , Pericytes: Developmental, Physiological, and Pathological Perspectives, Problems, and Promises, Developmental Cell. (2011) 21, no. 2, 193–215, 10.1016/j.devcel.2011.07.001. 21839917

[8] Farahani R. M. and Xaymardan M. , Platelet-Derived Growth Factor Receptor Alpha as a Marker of Mesenchymal Stem Cells in Development and Stem Cell Biology, Stem Cells International. (2015) 2015, 10.1155/2015/362753, 362753. PMC4519552 26257789

[9] Hellström M. , Kalén M. , Lindahl P. , Abramsson A. , and Betsholtz C. , Role of PDGF-B and PDGFR-Beta in Recruitment of Vascular Smooth Muscle Cells and Pericytes During Embryonic Blood Vessel Formation in the Mouse, Development. (1999) 126, no. 14, 3047–3055, 10.1242/dev.126.14.3047. 10375497

[10] Armulik A. , Genové G. , and Mäe M. , et al.Pericytes Regulate the Blood-Brain Barrier, Nature. (2010) 468, no. 7323, 557–561, 10.1038/nature09522. 20944627

[11] Crisan M. , Yap S. , and Casteilla L. , et al.A Perivascular Origin for Mesenchymal Stem Cells in Multiple Human Organs, Cell Stem Cell. (2008) 3, no. 3, 301–313, 10.1016/j.stem.2008.07.003. 18786417

[12] Kunisaki Y. , Bruns I. , and Scheiermann C. , et al.Arteriolar Niches Maintain Haematopoietic Stem Cell Quiescence, Nature. (2013) 502, no. 7473, 637–643, 10.1038/nature12612. PMC3821873 24107994

[13] Stark K. , Eckart A. , and Haidari S. , et al.Capillary and Arteriolar Pericytes Attract Innate Leukocytes Exiting Through Venules and ’Instruct’ Them With Pattern-Recognition and Motility Programs, Nature Immunology. (2013) 14, no. 1, 41–51, 10.1038/ni.2477. 23179077

[14] Ding L. , Saunders T. L. , Enikolopov G. , and Morrison S. J. , Endothelial and Perivascular Cells Maintain Haematopoietic Stem Cells, Nature. (2012) 481, no. 7382, 457–462, 10.1038/nature10783. PMC3270376 22281595

[15] Morrison S. J. and Spradling A. C. , Stem Cells and Niches: Mechanisms That Promote Stem Cell Maintenance Throughout Life, Cell. (2008) 132, no. 4, 598–611, 10.1016/j.cell.2008.01.038. PMC4505728 18295578

[16] Scadden D. T. , The Stem-Cell Niche as an Entity of Action, Nature. (2006) 441, no. 7097, 1075–1079, 10.1038/nature04957. 16810242

[17] Li L. and Clevers H. , Coexistence of Quiescent and Active Adult Stem Cells in Mammals, Science. (2010) 327, no. 5965, 542–545, 10.1126/science.1180794. PMC4105182 20110496

[18] Bjarnegård M. , Enge M. , and Norlin J. , et al.Endothelium-Specific Ablation of PDGFB Leads to Pericyte Loss and Glomerular, Cardiac and Placental Abnormalities, Development. (2004) 131, no. 8, 1847–1857, 10.1242/dev.01080. 15084468

[19] Hall C. N. , Reynell C. , and Gesslein B. , et al.Capillary Pericytes Regulate Cerebral Blood Flow in Health and Disease, Nature. (2014) 508, no. 7494, 55–60, 10.1038/nature13165. PMC3976267 24670647

[20] Hu J. , Hameed M. R. , and Agaram N. P. , et al.PDGFRβ Signaling Cooperates With β-Catenin to Modulate c-Abl and Biologic Behavior of Desmoid-Type Fibromatosis, Clinical Cancer Research. (2024) 30, no. 2, 450–461, 10.1158/1078-0432.CCR-23-2313. PMC10792363 37943631

[21] Bonner J. C. , Regulation of PDGF and Its Receptors in Fibrotic Diseases, Cytokine & Growth Factor Reviews. (2004) 15, no. 4, 255–273, 10.1016/j.cytogfr.2004.03.006. 15207816

[22] Discher D. E. , Janmey P. , and Wang Y.-L. , Tissue Cells Feel and Respond to the Stiffness of Their Substrate, Science. (2005) 310, no. 5751, 1139–1143, 10.1126/science.1116995. 16293750

[23] Pierce G. F. , Mustoe T. A. , and Lingelbach J. , et al.Platelet-Derived Growth Factor and Transforming Growth Factor-Beta Enhance Tissue Repair Activities by Unique Mechanisms, The Journal of Cell Biology. (1989) 109, no. 1, 429–440, 10.1083/jcb.109.1.429. PMC2115493 2745556

[24] Uutela M. , Wirzenius M. , and Paavonen K. , et al.PDGF-D Induces Macrophage Recruitment, Increased Interstitial Pressure, and Blood Vessel Maturation During Angiogenesis, Blood. (2004) 104, no. 10, 3198–3204, 10.1182/blood-2004-04-1485. 15271796

[25] Richardson T. P. , Peters M. C. , Ennett A. B. , and Mooney D. J. , Polymeric System for Dual Growth Factor Delivery, Nature Biotechnology. (2001) 19, no. 11, 1029–1034, 10.1038/nbt1101-1029. 11689847

[26] Trojanowska M. , Role of PDGF in Fibrotic Diseases and Systemic Sclerosis, Rheumatology (Oxford). (2008) 47, no. Suppl 5, v2–v4. 10.1093/rheumatology/ken265 18784131

[27] Gilbert P. M. , Havenstrite K. L. , and Magnusson K. E. G. , et al.Substrate Elasticity Regulates Skeletal Muscle Stem Cell Self-Renewal in Culture, Science. (2010) 329, no. 5995, 1078–1081, 10.1126/science.1191035. PMC2929271 20647425

[28] Zhang J. , Niu C. , and Ye L. , et al.Identification of the Haematopoietic Stem Cell Niche and Control of the Niche Size, Nature. (2003) 425, no. 6960, 836–841, 10.1038/nature02041. 14574412

[29] Chhabra A. , Lechner A. J. , and Ueno M. , et al.Trophoblasts Regulate the Placental Hematopoietic Niche Through PDGF-B Signaling, Developmental Cell. (2012) 22, no. 3, 651–659, 10.1016/j.devcel.2011.12.022. PMC3395466 22387002

[30] Greenwald A. C. , Licht T. , and Kumar S. , et al.VEGF Expands Erythropoiesis via Hypoxia-Independent Induction of Erythropoietin in Noncanonical Perivascular Stromal Cells, Journal of Experimental Medicine. (2019) 216, no. 1, 215–230, 10.1084/jem.20180752. PMC6314526 30545903

[31] Lim S.-E. , Esain V. , and Kwan W. , et al.HIF1α-Induced PDGFRβ Signaling Promotes Developmental HSC Production via IL-6 Activation, Experimental Hematology. (2017) 46, 83–95.e6, 10.1016/j.exphem.2016.10.002. PMC5338611 27751871

[32] Kusumbe A. P. , Ramasamy S. K. , and Itkin T. , et al.Age-Dependent Modulation of Vascular Niches for Haematopoietic Stem Cells, Nature. (2016) 532, no. 7599, 380–384, 10.1038/nature17638. PMC5035541 27074508

[33] Medves S. , Noel L. A. , and Montano-Almendras C. P. , et al.Multiple Oligomerization Domains of KANK1-PDGFRβ are Required for JAK2-Independent Hematopoietic Cell Proliferation and Signaling via STAT5 and ERK, Haematologica. (2011) 96, no. 10, 1406–1414, 10.3324/haematol.2011.040147. PMC3186300 21685469

[34] Atfi A. , Prunier C. , and Mazars A. , et al.The Oncogenic TEL/PDGFR Beta Fusion Protein Induces Cell Death Through JNK/SAPK Pathway, Oncogene. (1999) 18, no. 26, 3878–3885, 10.1038/sj.onc.1202734. 10445851

[35] Lee E. and Rewolinski D. , Evaluation of CXCR4 Inhibition in the Prevention and Intervention Model of Laser-Induced Choroidal Neovascularization, Investigative Opthalmology & Visual Science. (2010) 51, no. 7, 3666–3672, 10.1167/iovs.09-3802. 20042641

[36] Fierro F. , Illmer T. , and Jing D. , et al.Inhibition of Platelet-Derived Growth Factor Receptorbeta by Imatinib Mesylate Suppresses Proliferation and Alters Differentiation of Human Mesenchymal Stem Cells In Vitro, Cell Proliferation. (2007) 40, no. 3, 355–366, 10.1111/j.1365-2184.2007.00438.x. PMC6496321 17531080

[37] Bandeira D. S. , Kilpatrick A. M. , and Marques M. , et al.PDGFRβ(+) Cells Play a Dual Role as Hematopoietic Precursors and Niche Cells During Mouse Ontogeny, Cell Reports. (2022) 40, no. 3, 10.1016/j.celrep.2022.111114, 111114. PMC9638014 35858557

[38] Prasad P. and Cancelas J. A. , From Marrow to Bone and Fat: Exploring the Multifaceted Roles of Leptin Receptor Positive Bone Marrow Mesenchymal Stromal Cells, Cells. (2024) 13, no. 11, 10.3390/cells13110910, 910. PMC11171870 38891042

[39] Menezes K. , Rosa B. G. , and Freitas C. , et al.Human Mesenchymal Stromal/Stem Cells Recruit Resident Pericytes and Induce Blood Vessels Maturation to Repair Experimental Spinal Cord Injury in Rats, Scientific Reports. (2020) 10, no. 1, 10.1038/s41598-020-76290-0, 19604. PMC7658254 33177535

[40] Lin R.-Z. , Moreno-Luna R. , Li D. , Jaminet S.-C. , Greene A. K. , and Melero-Martin J. M. , Human Endothelial Colony-Forming Cells Serve as Trophic Mediators for Mesenchymal Stem Cell Engraftment via Paracrine Signaling, Proceedings of the National Academy of Sciences. (2014) 111, no. 28, 10137–10142, 10.1073/pnas.1405388111. PMC4104912 24982174

[41] Shaw I. W. , O’Sullivan E. D. , and Pisco A. O. , et al.Aging Modulates the Effects of Ischemic Injury Upon Mesenchymal Cells Within the Renal Interstitium and Microvasculature, Stem Cells Translational Medicine. (2021) 10, no. 8, 1232–1248, 10.1002/sctm.20-0392. PMC8284778 33951342

[42] Nam H. , Kim G.-H. , and Bae Y.-K. , et al.Angiogenic Capacity of Dental Pulp Stem Cell Regulated by SDF-1α-CXCR4 Axis, Stem Cells International. (2017) 2017, 10.1155/2017/8085462, 8085462. PMC5447288 28588623

[43] Cousins F. L. , Filby C. E. , and Gargett C. E. , Endometrial Stem/Progenitor Cells-Their Role in Endometrial Repair and Regeneration, Frontiers in Reproductive Health. (2022) 3, 10.3389/frph.2021.811537, 811537. PMC9580754 36304009

[44] Chandran R. R. , Xie Y. , and Gallardo-Vara E. , et al.Distinct Roles of KLF4 in Mesenchymal Cell Subtypes During Lung Fibrogenesis, Nature Communications. (2021) 12, no. 1, 10.1038/s41467-021-27499-8, 7179. PMC8664937 34893592

[45] Kirkwood P. M. , Gibson D. A. , and Smith J. R. , et al.Single-Cell RNA Sequencing Redefines the Mesenchymal Cell Landscape of Mouse Endometrium, The FASEB Journal. (2021) 35, no. 4, 10.1096/fj.202002123R. PMC9328940 33710643

[46] Slukvin I. I. and Kumar A. , The Mesenchymoangioblast, Mesodermal Precursor for Mesenchymal and Endothelial Cells, Cellular and Molecular Life Sciences. (2018) 75, no. 19, 3507–3520, 10.1007/s00018-018-2871-3. PMC6328351 29992471

[47] Rajkumar V. S. , Shiwen X. , and Bostrom M. , et al.Platelet-Derived Growth Factor-Beta Receptor Activation is Essential for Fibroblast and Pericyte Recruitment During Cutaneous Wound Healing, The American Journal of Pathology. (2006) 169, no. 6, 2254–2265, 10.2353/ajpath.2006.060196. PMC1762470 17148686

[48] Tokunaga A. , Oya T. , and Ishii Y. , et al.PDGF Receptor Beta is a Potent Regulator of Mesenchymal Stromal Cell Function, Journal of Bone and Mineral Research. (2008) 23, no. 9, 1519–1528, 10.1359/jbmr.080409. 18410236

[49] Wei S.-T. , Huang Y.-C. , and Hsieh M.-L. , et al.Atypical Chemokine Receptor ACKR3/CXCR7 Controls Postnatal Vasculogenesis and Arterial Specification by Mesenchymal Stem Cells via Notch Signaling, Cell Death & Disease. (2020) 11, no. 5, 10.1038/s41419-020-2512-2, 307. PMC7198625 32366833

[50] Wang S. , Mo M. , and Wang J. , et al.Platelet-Derived Growth Factor Receptor Beta Identifies Mesenchymal Stem Cells With Enhanced Engraftment to Tissue Injury and Pro-Angiogenic Property, Cellular and Molecular Life Sciences. (2018) 75, no. 3, 547–561, 10.1007/s00018-017-2641-7. PMC11105282 28929173

[51] Sivaraj K. K. , Jeong H.-W. , and Dharmalingam B. , et al.Regional Specialization and Fate Specification of Bone Stromal Cells in Skeletal Development, Cell Reports. (2021) 36, no. 2, 10.1016/j.celrep.2021.109352, 109352. PMC8293626 34260921

[52] Periasamy R. , Elshaer S. L. , and Gangaraju R. , CD140b (PDGFRβ) Signaling in Adipose-Derived Stem Cells Mediates Angiogenic Behavior of Retinal Endothelial Cells, Regenerative Engineering and Translational Medicine. (2019) 5, no. 1, 1–9, 10.1007/s40883-018-0068-9. PMC6453132 30976657

[53] He S. , Hou T. , and Zhou J. , et al.Endothelial Cells Promote Migration of Mesenchymal Stem Cells via PDGF-BB/PDGFRβ-Src-Akt in the Context of Inflammatory Microenvironment Upon Bone Defect, Stem Cells International. (2022) 2022, 10.1155/2022/2401693, 2401693. PMC9526552 36193255

[54] Kitahashi T. , Kogawa R. , Nakamura K. , and Sekiya I. , Integrin β1, PDGFRβ, and Type II Collagen are Essential for Meniscus Regeneration by Synovial Mesenchymal Stem Cells in Rats, Scientific Reports. (2022) 12, no. 1, 10.1038/s41598-022-18476-2, 14148. PMC9391488 35986079

[55] Guo T. , Pei F. , and Zhang M. , et al.Vascular Architecture Regulates Mesenchymal Stromal Cell Heterogeneity via P53-PDGF Signaling in the Mouse Incisor, Cell Stem Cell. (2024) 31, no. 6, 904–920.e6, 10.1016/j.stem.2024.04.011. PMC11162319 38703771

[56] Veevers-Lowe J. , Ball S. G. , Shuttleworth A. , and Kielty C. M. , Mesenchymal Stem Cell Migration is Regulated by Fibronectin Through α5β1-Integrin-Mediated Activation of PDGFR-β and Potentiation of Growth Factor Signals, Journal of Cell Science. (2011) 124, no. 8, 1288–1300, 10.1242/jcs.076935. PMC3065385 21429937

[57] Murray I. R. , Gonzalez Z. N. , and Baily J. , et al.αv Integrins on Mesenchymal Cells Regulate Skeletal and Cardiac Muscle Fibrosis, Nature Communications. (2017) 8, no. 1, 10.1038/s41467-017-01097-z, 1118. PMC5653645 29061963

[58] Aomatsu E. , Chosa N. , Nishihira S. , Sugiyama Y. , Miura H. , and Ishisaki A. , Cell-Cell Adhesion Through N-Cadherin Enhances VCAM-1 Expression via PDGFRβ in a Ligand-Independent Manner in Mesenchymal Stem Cells, International Journal of Molecular Medicine. (2014) 33, no. 3, 565–572, 10.3892/ijmm.2013.1607. 24378362

[59] Fang J. , Huang X. , and Han X. , et al.Endothelial Progenitor Cells Promote Viability and Nerve Regenerative Ability of Mesenchymal Stem Cells Through PDGF-BB/PDGFR-β Signaling, Aging. (2020) 12, no. 1, 106–121, 10.18632/aging.102604. PMC6977666 31899688

[60] Supakul S. , Yao K. , and Ochi H. , et al.Pericytes as a Source of Osteogenic Cells in Bone Fracture Healing, International Journal of Molecular Sciences. (2019) 20, no. 5, 10.3390/ijms20051079, 1079. PMC6429337 30832329

[61] James A. W. , Hindle P. , and Murray I. R. , et al.Pericytes for the Treatment of Orthopedic Conditions, Pharmacology & Therapeutics. (2017) 171, 93–103, 10.1016/j.pharmthera.2016.08.003. PMC6434715 27510330

[62] Li L. , Lu X. , Lian Q. , Wang X. , Jia C. , and Xu C. , Apoptotic Vesicles of Mesenchymal Stem Cells Promote M2 Polarization and Alleviate Early-Onset Preeclampsia via miR-191-5p, Stem Cell Research & Therapy. (2025) 16, no. 1, 10.1186/s13287-025-04546-5, 414. PMC12312347 40739581

[63] Doetsch F. , A Niche for Adult Neural Stem Cells, Current Opinion in Genetics & Development. (2003) 13, no. 5, 543–550, 10.1016/j.gde.2003.08.012. 14550422

[64] Fuentealba L. C. , Obernier K. , and Alvarez-Buylla A. , Adult Neural Stem Cells Bridge Their Niche, Cell Stem Cell. (2012) 10, no. 6, 698–708, 10.1016/j.stem.2012.05.012. PMC3726005 22704510

[65] Bond A M. , Ming G.-L. , and Song H. , Adult Mammalian Neural Stem Cells and Neurogenesis: Five Decades Later, Cell Stem Cell. (2015) 17, no. 4, 385–395, 10.1016/j.stem.2015.09.003. PMC4683085 26431181

[66] Lim D. A. and Alvarez-Buylla A. , Adult Neural Stem Cells Stake Their Ground, Trends in Neurosciences. (2014) 37, no. 10, 563–571, 10.1016/j.tins.2014.08.006. PMC4203324 25223700

[67] King N. E. , Courtney J.-M. , and Brown L. S. , et al.Induced Pluripotent Stem Cell Derived Pericytes Respond to Mediators of Proliferation and Contractility, Stem Cell Research & Therapy. (2024) 15, no. 1, 10.1186/s13287-024-03671-x, 59. PMC10910734 38433209

[68] Kaushik G. , Gupta K. , and Harms V. , et al.Engineered Perineural Vascular Plexus for Modeling Developmental Toxicity, Advanced Healthcare Materials. (2020) 9, no. 16. 10.1002/adhm.202000825 PMC8016604 32613760

[69] Lui J. H. , Nowakowski T. J. , Pollen A. A. , Javaherian A. , Kriegstein A. R. , and Oldham M. C. , Radial Glia Require PDGFD-PDGFRβ Signalling in Human but Not Mouse Neocortex, Nature. (2014) 515, no. 7526, 264–268, 10.1038/nature13973. PMC4231536 25391964

[70] Nakata M. , Nakagomi T. , Maeda M. , Nakano-Doi A. , Momota Y. , and Matsuyama T. , Induction of Perivascular Neural Stem Cells and Possible Contribution to Neurogenesis Following Transient Brain Ischemia/Reperfusion Injury, Translational Stroke Research. (2017) 8, no. 2, 131–143, 10.1007/s12975-016-0479-1. 27352866

[71] Sato H. , Ishii Y. , and Yamamoto S. , et al.PDGFR-β Plays a Key Role in the Ectopic Migration of Neuroblasts in Cerebral Stroke, Stem Cells. (2016) 34, no. 3, 685–698, 10.1002/stem.2212. 26435273

[72] Tan Z. , Xiao L. , and Ma J. , et al.Integrating Hydrogels Manipulate ECM Deposition After Spinal Cord Injury for Specific Neural Reconnections via Neuronal Relays, Science Advances. (2024) 10, no. 27, 10.1126/sciadv.ado9120. PMC11221524 38959311

[73] Srinivasan A. , Chang S.-Y. , Zhang S. , Toh W. S. , and Toh Y.-C. , Substrate Stiffness Modulates the Multipotency of Human Neural Crest Derived Ectomesenchymal Stem Cells via CD44 Mediated PDGFR Signaling, Biomaterials. (2018) 167, 153–167, 10.1016/j.biomaterials.2018.03.022. 29571051

[74] Ogura S. , Kurata K. , and Hattori Y. , et al.Sustained Inflammation After Pericyte Depletion Induces Irreversible Blood-Retina Barrier Breakdown, JCI Insight. (2017) 2, no. 3, 10.1172/jci.insight.90905. PMC5291729 28194443

[75] Delgado A. C. , Maldonado-Soto A. R. , and Silva-Vargas V. , et al.Release of Stem Cells From Quiescence Reveals Gliogenic Domains in the Adult Mouse Brain, Science. (2021) 372, no. 6547, 1205–1209, 10.1126/science.abg8467. 34112692

[76] Xu G. , Shen J. , and Ishii Y. , et al.Functional Analysis of Platelet-Derived Growth Factor Receptor-β in Neural Stem/Progenitor Cells, Neuroscience. (2013) 238, 195–208, 10.1016/j.neuroscience.2013.02.021. 23454370

[77] Chen Q. , Song H. , and Liu C. , et al.The Interaction of EphA4 With PDGFRβ Regulates Proliferation and Neuronal Differentiation of Neural Progenitor Cells In Vitro and Promotes Neurogenesis In Vivo, Frontiers in Aging Neuroscience. (2020) 12, 10.3389/fnagi.2020.00007, 7. PMC7026009 32116646

[78] Li H.-H. , Liu Y. , and Chen H.-S. , et al.PDGF-BB-Dependent Neurogenesis Buffers Depressive-Like Behaviors by Inhibition of GABAergic Projection From Medial Septum to Dentate Gyrus, Advanced Science. (2023) 10, no. 22, 10.1002/advs.202301110. PMC10401107 37325895

[79] Janowski M. , Lukomska B. , and Domanska-Janik K. , Migratory Capabilities of Human Umbilical Cord Blood-Derived Neural Stem Cells (HUCB-NSC) In Vitro, Acta Neurobiologiae Experimentalis. (2011) 71, no. 1, 24–35, 10.55782/ane-2011-1820. 21499324

[80] Pei D. , Liu N. , and Li D. , et al.Inhibition of Platelet-Derived Growth Factor Receptor β Reduces Reactive Glia and Scar Formation After Traumatic Brain Injury in Mice, Brain Research Bulletin. (2017) 134, 121–127, 10.1016/j.brainresbull.2017.06.020. 28684344

[81] Morita T. , Sasaki M. , and Kataoka-Sasaki Y. , et al.Intravenous Infusion of Mesenchymal Stem Cells Promotes Functional Recovery in a Model of Chronic Spinal Cord Injury, Neuroscience. (2016) 335, 221–231, 10.1016/j.neuroscience.2016.08.037. 27586052

[82] Wu W. , Jia S. , and Xu H. , et al.Supramolecular Hydrogel Microspheres of Platelet-Derived Growth Factor Mimetic Peptide Promote Recovery From Spinal Cord Injury, ACS Nano. (2023) 17, no. 4, 3818–3837, 10.1021/acsnano.2c12017. 36787636

[83] Galvez-Contreras A. Y. , Gonzalez-Castaneda R. E. , and Luquin S. , et al.Diphenylhydantoin Promotes Proliferation in the Subventricular Zone and Dentate Gyrus, American Journal of Neuroscience. (2012) 3, no. 1, 1–9, 10.3844/amjnsp.2012.1.9. PMC3902771 24478822

[84] Adile A. A. , Bakhshinyan D. , and Suk Y. , et al.An Effective Kinase Inhibition Strategy for Metastatic Recurrent Childhood Medulloblastoma, Journal of Neuro-Oncology. (2023) 163, no. 3, 635–645, 10.1007/s11060-023-04372-w. 37354357

[85] Gimple R. C. , Zhang G. , and Wang S. , et al.Sorting Nexin 10 Sustains PDGF Receptor Signaling in Glioblastoma Stem Cells via Endosomal Protein Sorting, JCI Insight. (2023) 8, no. 6, 10.1172/jci.insight.158077. PMC10070110 36795488

[86] Dolma S. , Selvadurai H. J. , and Lan X. , et al.Inhibition of Dopamine Receptor D4 Impedes Autophagic Flux, Proliferation, and Survival of Glioblastoma Stem Cells, Cancer Cell. (2016) 29, no. 6, 859–873, 10.1016/j.ccell.2016.05.002. PMC5968455 27300435

[87] Li W. , Hayashida Y. , Chen Y.-T. , and Tseng S. C. G. , Niche Regulation of Corneal Epithelial Stem Cells at the Limbus, Cell Research. (2007) 17, no. 1, 26–36, 10.1038/sj.cr.7310137. PMC3190132 17211449

[88] Farrelly O. , Suzuki-Horiuchi Y. , and Brewster M. , et al.Two-Photon Live Imaging of Single Corneal Stem Cells Reveals Compartmentalized Organization of the Limbal Niche, Cell Stem Cell. (2021) 28, no. 7, 1233–1247.e4, 10.1016/j.stem.2021.02.022. PMC8559309 33984283

[89] Li S. , Sun H. , Chen L. , and Fu Y. , Targeting Limbal Epithelial Stem Cells: Master Conductors of Corneal Epithelial Regeneration From the Bench to Multilevel Theranostics, Journal of Translational Medicine. (2024) 22, no. 1, 10.1186/s12967-024-05603-y, 794. PMC11350997 39198892

[90] Polisetti N. , Gießl A. , and Zenkel M. , et al.Melanocytes as Emerging Key Players in Niche Regulation of Limbal Epithelial Stem Cells, The Ocular Surface. (2021) 22, 172–189, 10.1016/j.jtos.2021.08.006. 34425298

[91] Mirmoeini K. , Tajdaran K. , and Zhang J. , et al.Schwann Cells are Key Regulators of Corneal Epithelial Renewal, Investigative Opthalmology & Visual Science. (2023) 64, no. 4, 10.1167/iovs.64.4.7, 7. PMC10103722 37036418

[92] Sun D. , Zhang X. , and Chen R. , et al.Decoding Cellular Plasticity and Niche Regulation of Limbal Stem Cells During Corneal Wound Healing, Stem Cell Research & Therapy. (2024) 15, no. 1, 10.1186/s13287-024-03816-y, 201. PMC11227725 38971839

[93] Altshuler A. , Amitai-Lange A. , and Tarazi N. , et al.Discrete Limbal Epithelial Stem Cell Populations Mediate Corneal Homeostasis and Wound Healing, Cell Stem Cell. (2021) 28, no. 7, 1248–1261.e8, 10.1016/j.stem.2021.04.003. PMC8254798 33984282

[94] Guo P. , Sun H. , and Zhang Y. , et al.Limbal Niche Cells are a Potent Resource of Adult Mesenchymal Progenitors, Journal of Cellular and Molecular Medicine. (2018) 22, no. 7, 3315–3322, 10.1111/jcmm.13635. PMC6010802 29679460

[95] Wang W. , Li S. , and Xu L. , et al.Differential Gene Expression Between Limbal Niche Progenitors and Bone Marrow Derived Mesenchymal Stem Cells, International Journal of Medical Sciences. (2020) 17, no. 4, 549–557, 10.7150/ijms.40881. PMC7053302 32174786

[96] Li G. , Zhang Y. , and Cai S. , et al.Human Limbal Niche Cells are a Powerful Regenerative Source for the Prevention of Limbal Stem Cell Deficiency in a Rabbit Model, Scientific Reports. (2018) 8, no. 1, 10.1038/s41598-018-24862-6, 6566. PMC5919904 29700361

[97] Su G. , Guo X. , and Xu L. , et al.Isolation and Characterization of Rabbit Limbal Niche Cells, Experimental Eye Research. (2024) 241, 10.1016/j.exer.2024.109838, 109838. 38395213

[98] Zhu H. , Wang W. , and Tan Y. , et al.Limbal Niche Cells and Three-Dimensional Matrigel-Induced Dedifferentiation of Mature Corneal Epithelial Cells, Investigative Opthalmology & Visual Science. (2022) 63, no. 5, 10.1167/iovs.63.5.1, 1. PMC9078055 35499835

[99] Shen J. , Wang W. , and Chen L. , et al.SIRT1 Alleviates Hyperglycemia-Induced Oxidative Stress and Dysfunction of Limbal Niche Cells by Deacetylating FOXO1, Free Radical Biology and Medicine. (2025) 239, 130–144, 10.1016/j.freeradbiomed.2025.07.032. 40714155

[100] Meshko B. , Volatier T. L. A. , and Hadrian K. , et al.ABCB5+ Limbal Epithelial Stem Cells Inhibit Developmental but Promote Inflammatory (Lymph) Angiogenesis While Preventing Corneal Inflammation, Cells. (2023) 12, no. 13, 10.3390/cells12131731, 1731. PMC10341195 37443766

[101] Mei H. , Gonzalez S. , and Deng S. , Extracellular Matrix is an Important Component of Limbal Stem Cell Niche, Journal of Functional Biomaterials. (2012) 3, no. 4, 879–894, 10.3390/jfb3040879. PMC4030928 24955751

[102] Polisetti N. , Sorokin L. , and Okumura N. , et al.Laminin-511 and -521-Based Matrices for Efficient Ex Vivo-Expansion of Human Limbal Epithelial Progenitor Cells, Scientific Reports. (2017) 7, no. 1, 10.1038/s41598-017-04916-x, 5152. PMC5506065 28698551

[103] Bhattacharya S. , Mukherjee A. , and Pisano S. , et al.The Biophysical Property of the Limbal Niche Maintains Stemness Through YAP, Cell Death & Differentiation. (2023) 30, no. 6, 1601–1614, 10.1038/s41418-023-01156-7. PMC10244376 37095157

[104] Zhou X. , Xu L. , Tan Y. , Wang W. , Huang X. , and Li G. , Stiffness Regulates the Morphology and Stemness of Limbal Niche Cells Through Unique nYAP/cYAP Translocation, Investigative Ophthalmology & Visual Science. (2025) 66, no. 2, 43–43, 10.1167/iovs.66.2.43. PMC11824500 39951297

[105] Xie H.-T. , Chen S.-Y. , Li G.-G. , and Tseng S. C. G. , Limbal Epithelial Stem/Progenitor Cells Attract Stromal Niche Cells by SDF-1/CXCR4 Signaling to Prevent Differentiation, Stem Cells. (2011) 29, no. 11, 1874–1885, 10.1002/stem.743. 21948620

[106] Han B. , Chen S.-Y. , Zhu Y.-T. , and Tseng S. C. G. , Integration of BMP/Wnt Signaling to Control Clonal Growth of Limbal Epithelial Progenitor Cells by Niche Cells, Stem Cell Research. (2014) 12, no. 2, 562–573, 10.1016/j.scr.2014.01.003. PMC3952206 24530980

[107] Zhu L. , Zhang W. , and Zhu J. , et al.Cotransplantation of Limbal Epithelial and Stromal Cells for Ocular Surface Reconstruction, Ophthalmology Science. (2022) 2, no. 2, 10.1016/j.xops.2022.100148, 100148. PMC9560570 36249679

[108] Zhou T. , Huang X. , and Tan Y. , et al.Limbal Niche Cell-Derived Exosomes Accelerate Corneal Epithelial Repair Through PI3K/Akt Activation and FOXO3 Inhibition, Experimental Eye Research. (2026) 269, 10.1016/j.exer.2026.111049, 111049. 42119839

[109] Afsharkhamseh N. , Movahedan A. , and Gidfar S. , et al.Stability of Limbal Stem Cell Deficiency After Mechanical and Thermal Injuries in Mice, Experimental Eye Research. (2016) 145, 88–92, 10.1016/j.exer.2015.11.012. PMC4979604 26607808

[110] Leszczynska A. , Kulkarni M. , Ljubimov A. V. , and Saghizadeh M. , Exosomes From Normal and Diabetic Human Corneolimbal Keratocytes Differentially Regulate Migration, Proliferation and Marker Expression of Limbal Epithelial Cells, Scientific Reports. (2018) 8, no. 1, 10.1038/s41598-018-33169-5, 15173. PMC6182003 30310159

[111] Sánchez-Lanzas R. A. , Kalampalika F. , and Ganuza M. , Diversity in the Bone Marrow Niche: Classic and Novel Strategies to Uncover Niche Composition, British Journal of Haematology. (2022) 199, no. 5, 647–664, 10.1111/bjh.18355. PMC9796334 35837798

[112] Mangialardi G. , Cordaro A. , and Madeddu P. , The Bone Marrow Pericyte: An Orchestrator of Vascular Niche, Regenerative Medicine. (2016) 11, no. 8, 883–895, 10.2217/rme-2016-0121. PMC5677781 27885901

[113] Wilson C. L. , Stephenson S. E. , Higuero J. P. , Feghali-Bostwick C. , Hung C. F. , and Schnapp L. M. , Characterization of Human PDGFR-β-Positive Pericytes From IPF and Non-IPF Lungs, American Journal of Physiology-Lung Cellular and Molecular Physiology. (2018) 315, no. 6, L991–L1002, 10.1152/ajplung.00289.2018. PMC6337011 30335500

[114] Boulais P. E. and Frenette P. S. , Making Sense of Hematopoietic Stem Cell Niches, Blood. (2015) 125, no. 17, 2621–2629, 10.1182/blood-2014-09-570192. PMC4408288 25762174

[115] Kostallari E. , Baba-Amer Y. , and Alonso-Martin S. , et al.Pericytes in the Myovascular Niche Promote Post-Natal Myofiber Growth and Satellite Cell Quiescence, Development. (2015) 142, no. 7, 1242–1253, 10.1242/dev.115386. 25742797

[116] Sweeney M. and Foldes G. , It Takes Two: Endothelial-Perivascular Cell Cross-Talk in Vascular Development and Disease, Frontiers in Cardiovascular Medicine. (2018) 5, 10.3389/fcvm.2018.00154, 154. PMC6218412 30425990

[117] Layton T. B. , Williams L. , and Yang N. , et al.A Vasculature Niche Orchestrates Stromal Cell Phenotype Through PDGF Signaling: Importance in Human Fibrotic Disease, Proceedings of the National Academy of Sciences. (2022) 119, no. 13, 10.1073/pnas.2120336119. PMC9060460 35320046

[118] Chen J. , Luo Y. , and Huang H. , et al.CD146 is Essential for PDGFRβ-Induced Pericyte Recruitment, Protein & Cell. (2018) 9, no. 8, 743–747, 10.1007/s13238-017-0484-5. PMC6053352 29039032

[119] Song S. , Ewald A. J. , Stallcup W. , Werb Z. , and Bergers G. , PDGFRβ+ Perivascular Progenitor Cells in Tumours Regulate Pericyte Differentiation and Vascular Survival, Nature Cell Biology. (2005) 7, no. 9, 870–879, 10.1038/ncb1288. PMC2771163 16113679

[120] Özen I. , Boix J. , and Paul G. , Perivascular Mesenchymal Stem Cells in the Adult Human Brain: A Future Target for Neuroregeneration?, Clinical and Translational Medicine. (2012) 1, no. 1, 10.1186/2001-1326-1-30, 30. PMC3561038 23369339

[121] Mills S. , Cowin A. , and Kaur P. , Pericytes, Mesenchymal Stem Cells and the Wound Healing Process, Cells. (2013) 2, no. 3, 621–634, 10.3390/cells2030621. PMC3972668 24709801

[122] Kelly-Goss M. R. , Sweat R. S. , Stapor P. C. , Peirce S. M. , and Murfee W. L. , Targeting Pericytes for Angiogenic Therapies, Microcirculation. (2014) 21, no. 4, 345–357, 10.1111/micc.12107. PMC4079092 24267154

[123] Lim H. K. , Periasamy P. , and O’Neill H. C. , In Vitro Murine Hematopoiesis Supported by Signaling From a Splenic Stromal Cell Line, Stem Cells International. (2018) 2018, 10.1155/2018/9896142, 9896142. PMC6323497 30675170

[124] Gargett C. E. , Schwab K. E. , and Deane J. A. , Endometrial Stem/Progenitor Cells: The First 10 Years, Human Reproduction Update. (2016) 22, no. 2, 137–163, 10.1093/humupd/dmv051. PMC4755439 26552890

[125] Helbling P. M. , Piñeiro-Yáñez E. , and Gerosa R. , et al.Global Transcriptomic Profiling of the Bone Marrow Stromal Microenvironment During Postnatal Development, Aging, and Inflammation, Cell Reports. (2019) 29, no. 10, 3313–3330.e4, 10.1016/j.celrep.2019.11.004. 31801092

[126] Liu Y. , Lei P. , Row S. , and Andreadis S. T. , Cadherin-11 Binds to PDGFRβ and Enhances Cell Proliferation and Tissue Regeneration via the PDGFR-AKT Signaling Axis, The FASEB Journal. (2020) 34, no. 3, 3792–3804, 10.1096/fj.201902613R. 31930567

[127] Camorani S. , Hill B. S. , and Fontanella R. , et al.Inhibition of Bone Marrow-Derived Mesenchymal Stem Cells Homing Towards Triple-Negative Breast Cancer Microenvironment Using an Anti-PDGFRβ Aptamer, Theranostics. (2017) 7, no. 14, 3595–3607, 10.7150/thno.18974. PMC5596446 28912898

[128] Wang H.-H. , Cui Y.-L. , and Zaorsky N. G. , et al.Mesenchymal Stem Cells Generate Pericytes to Promote Tumor Recurrence via Vasculogenesis After Stereotactic Body Radiation Therapy, Cancer Letters. (2016) 375, no. 2, 349–359, 10.1016/j.canlet.2016.02.033. 26996301

[129] de Faria F. W. , Riedel N. C. , and Münter D. , et al.ETMR Stem-Like State and Chemo-Resistance Are Supported by Perivascular Cells at Single-Cell Resolution, Nature Communications. (2025) 16, no. 1, 10.1038/s41467-025-60442-9, 5394. PMC12198369 40562749

[130] Gaengel K. , Genové G. , Armulik A. , and Betsholtz C. , Endothelial-Mural Cell Signaling in Vascular Development and Angiogenesis, Arteriosclerosis, Thrombosis, and Vascular Biology. (2009) 29, no. 5, 630–638. 10.1161/ATVBAHA.107.161521 19164813

[131] Amin S. , Jalilian E. , and Katz E. , et al.The Limbal Niche and Regenerative Strategies, Vision. (2021) 5, no. 4, 10.3390/vision5040043, 43. PMC8544688 34698278

[132] Lebeau G. , Ah-Pine F. , and Daniel M. , et al.Perivascular Mesenchymal Stem/Stromal Cells, an Immune Privileged Niche for Viruses?, International Journal of Molecular Sciences. (2022) 23, no. 14, 10.3390/ijms23148038, 8038. PMC9317325 35887383

[133] Li G.-G. , Chen S.-Y. , Xie H.-T. , Zhu Y.-T. , and Tseng S. C. G. , Angiogenesis Potential of Human Limbal Stromal Niche Cells, Investigative Opthalmology & Visual Science. (2012) 53, no. 7, 3357–3367, 10.1167/iovs.11-9414. PMC3374622 22538425

[134] Funderburgh J. L. , Funderburgh M. L. , and Du Y. , Stem Cells in the Limbal Stroma, The Ocular Surface. (2016) 14, no. 2, 113–120, 10.1016/j.jtos.2015.12.006. PMC4842326 26804252

[135] Lim P. , Fuchsluger T. A. , and Jurkunas U. V. , Limbal Stem Cell Deficiency and Corneal Neovascularization, Seminars in Ophthalmology. (2009) 24, no. 3, 139–148, 10.1080/08820530902801478. 19437349

[136] Ljubimov A. V. and Saghizadeh M. , Progress in Corneal Wound Healing, Progress in Retinal and Eye Research. (2015) 49, 17–45, 10.1016/j.preteyeres.2015.07.002. PMC4651844 26197361

[137] Soleimani M. , Cheraqpour K. , Koganti R. , Baharnoori S. M. , and Djalilian A. R. , Concise Review: Bioengineering of Limbal Stem Cell Niche, Bioengineering. (2023) 10, no. 1, 10.3390/bioengineering10010111, 111. PMC9855097 36671683

[138] Gharibi B. , Ghuman M. S. , and Hughes F. J. , Akt- and Erk-Mediated Regulation of Proliferation and Differentiation During PDGFRβ-Induced MSC Self-Renewal, Journal of Cellular and Molecular Medicine. (2012) 16, no. 11, 2789–2801, 10.1111/j.1582-4934.2012.01602.x. PMC4118247 22805337

